# Feline Papillomatosis

**DOI:** 10.3390/v17010059

**Published:** 2025-01-02

**Authors:** Herman Egberink, Katrin Hartmann, Ralf Mueller, Maria Grazia Pennisi, Sándor Belák, Séverine Tasker, Karin Möstl, Diane D. Addie, Corine Boucraut-Baralon, Tadeusz Frymus, Regina Hofmann-Lehmann, Fulvio Marsilio, Etienne Thiry, Uwe Truyen, Margaret J. Hosie

**Affiliations:** 1 Department of Biomolecular Health Sciences, Faculty of Veterinary Medicine, University of Utrecht, 3584 CL Utrecht, The Netherlands; 2 Clinic of Small Animal Medicine, Centre for Clinical Veterinary Medicine, LMU Munich, 80539 Munich, Germany; hartmann@lmu.de (K.H.); r.mueller@lmu.de (R.M.); 3 I Periodeuti ASC, 89122 Reggio Calabria, Italy; mariagrazia.pennisi@unime.it; 4 Department of Biomedical Sciences and Veterinary Public Health (BVF), Swedish University of Agri-Cultural Sciences (SLU), P.O. Box 7036, 750 07 Uppsala, Sweden; sandor.belak@slu.se; 5 Bristol Veterinary School, University of Bristol, Bristol BS40 5DU, UK; s.tasker@bristol.ac.uk; 6 Mars Veterinary Health, Solihull B90 4BN, UK; 7 Retired from Institute of Virology, Department for Pathobiology, University of Veterinary Medicine, 1210 Vienna, Austria; karinmoestl@gmail.com; 8 Independent Researcher, 64000 Pyrénées Aquitaine, France; draddie@catvirus.com; 9 Scanelis Laboratory, 31770 Colomiers, France; corine.boucraut@scanelis.com; 10 Department of Small Animal Diseases with Clinic, Institute of Veterinary Medicine, Warsaw University of Life Sciences—SGGW, 02-787 Warsaw, Poland; tadeusz_frymus@sggw.edu.pl; 11 Clinical Laboratory, Department of Clinical Diagnostics and Services, Vetsuisse Faculty, University of Zurich, 8057 Zurich, Switzerland; rhofmann@vetclinics.uzh.ch; 12 Faculty of Veterinary Medicine, Università degli Studi di Teramo, 64100 Teramo, Italy; fmarsilio@unite.it; 13 Veterinary Virology and Animal Viral Diseases, Department of Infectious and Parasitic Diseases, FARAH Research Centre, Faculty of Veterinary Medicine, Liège University, B-4000 Liège, Belgium; etienne.thiry@uliege.be; 14 Institute of Animal Hygiene and Veterinary Public Health, University of Leipzig, 04103 Leipzig, Germany; truyen@vetmed.uni-leipzig.de; 15 MRC—University of Glasgow Centre for Virus Research, Glasgow G61 1QH, UK; margaret.hosie@glasgow.ac.uk

**Keywords:** papillomavirus, feline, FcaPV, papilloma, skin neoplasm

## Abstract

Different types of feline papillomaviruses (PVs) are associated with a variety of skin lesions and neoplasia, such as papillomas and cell carcinomas, but the virus can also be found in healthy skin. In this review, the European Advisory Board on Cat Diseases (ABCD), a scientifically independent board of veterinary experts on feline infectious diseases from 11 European Countries, discusses the current knowledge of feline PV infections. Cats most likely become infected through lesions or abrasions of the skin. Most PV infections remain asymptomatic. Besides cat-specific PVs, DNA sequences most closely related to human and bovine PVs have been detected in feline skin lesions. Diagnosis is supported by the histological detection of PV-induced cell changes and intralesional detection of viral antigen (immunostaining) or viral DNA (in situ hybridization). Immunostaining of p16CDKN2A protein (p16) can be performed as a proxy marker for PV-induced neoplasms. There is no specific treatment for PV-induced skin lesions. Spontaneous regression commonly occurs. In the case of invasive squamous cell carcinoma (ISCC), complete excision should be considered, if possible.

## 1. Introduction

Papillomaviruses (PVs) are epitheliotropic infectious agents that cause infections in humans as well as many different animal species, including fish. In general, PVs are host-species-specific with tropism for mucosal and cutaneous epithelia and a preference for specific locations on the body [1,2]. Most PVs cause asymptomatic or harmless infections, but some PV types can induce neoplasia. In each host, different PV types exist. In humans, more than 200 types have been identified [3]. Presently, eight different PV types with complete genome sequences have been identified in cats, seven feline PVs (Felis catus papillomaviruses 1-7; FcaPV1-7) and bovine PV14, which is a bovine-specific PV (BPV) found in cats with sarcoids [4,5,6]. In addition, several putative PVs, based on sequence similarities with known feline PVs, have been detected from lesions in cats [7,8]. This review presents an update on the current state of knowledge on infections with FcaPV.

## 2. Virus

**Figure 1:**
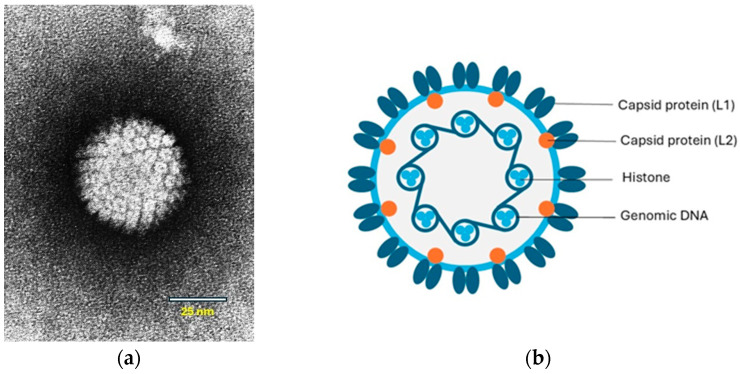
(**a**) Papilloma virion, negative contrast electron microscopic (EM) image. Source: Laboratory of Tumor Virus Biology, NIH-Visuals Online# AV-8610-3067. (**b**) Cross-sectional model of a papillomavirus. The circular dsDNA is associated with cellular histones and surrounded by a capsid consisting of the L1 and L2 proteins. © Karin de Lange (Aksent Veterinary Communications) for the ABCD.

Papillomaviruses are small viruses containing circular double-stranded DNA that belong to the family *Papillomaviridae* (Figure 1a,b).

The viral genome contains early (E) genes, which encode several E proteins, and two late (L) genes. Some of the E proteins play roles in viral transcription and replication. The E6 and E7 proteins are oncogenes that interfere with cell regulation. The L genes encode two structural proteins, L1 and L2, that form the capsid during virus assembly (Figure 1b). A third gene, L3, of which the function is unknown has only been identified in some papillomaviruses [9]. In human PV-induced neoplasms, the viral DNA is integrated into the host-cell DNA. However, in papillomas (warts), the viral DNA is not integrated and persists as an autonomously replicating episome. Viral DNA integration also appears to be uncommon within PV-induced neoplasms in animals [10]. However, in a recent study, genomic integration and expression of feline PV oncogenes were shown in feline Merkel cell carcinoma (MCC) in cats [11].

Two subfamilies of PVs are recognized: *firstpapillomavirinae* (containing 52 genera and several hundred species) and *secondpapillomavirinae* (currently one genus). The conserved L1 gene is used for the classification of PVs. If two PVs have less than 90% sequence similarity in the L gene, they are considered to be different types, and if less than 60% similarity, to be within different genera [12,13].

Feline PVs were previously designated as *Felis domesticus* PVs (FdPVs), but a more correct taxonomic name, Felis catus PVs (FcaPVs), has been proposed [14]. The feline PVs are classified within three genera: *Lambdapapillomavirus* (FcaPV1), *Dyothetapapillomavirus* (FcaPV2) and the *Taupapillomavirus* genus (FcaPV 3-7) [15]. The BPV, classified within the genus *Deltapapillomavirus,* is an exception to the rule that PVs are highly species-specific [4]. A novel FcaPV type was suggested in a cat with a cutaneous papilloma, based on a low level of sequence similarity with known FcaPVs [7] and another putative novel FcaPV type was determined in a feline osteoinductive squamous cell carcinoma [8].

## 3. Epidemiology

Papillomaviruses have been detected in several animal species and in humans as a cause of cutaneous lesions [16]. Although the viruses tend to be host-species-specific, sequences related to bovine and human PVs have been found in cats, suggesting cross-species transmission [17,18]. However, it is not excluded that the detection of human PVs might be due to cross-contamination during the sampling or processing of samples [4]. PV infection has also been detected in other felids, including the Florida panther subspecies of cougar (*Puma concolor coryi*), Bobcat (*Lynx rufus*), Asiatic lion (*Panthera leo persica*), Snow leopard (*Panthera unica*), clouded leopard (*Neofelis nebulosa*), cheetah (*Acinonyx jubatus*) and caracals (*Caracal caracal*) [19,20,21,22,23,24].

## 4. Pathogenesis

**Figure 2:**
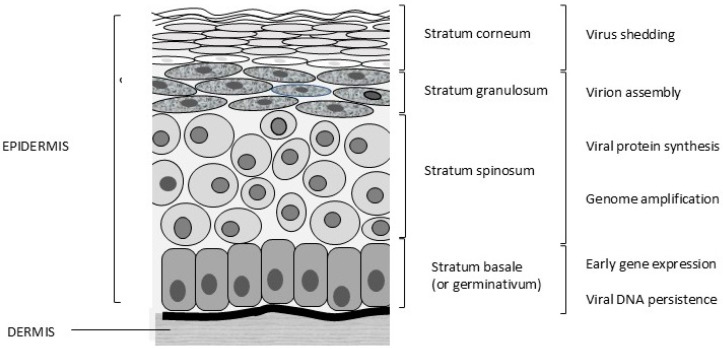
Different layers of feline skin and stages of the viral life cycle. © Karin de Lange (Aksent Veterinary Communications) for the ABCD.

Papillomaviruses are epitheliotropic infectious agents. Infections usually occur through lesions or abrasions of the skin. Initially, the basal cells of the stratum germinativum (stratum basale, Figure 2) are infected, which leads to hyperplasia and delayed maturation of cells in the stratum spinosum and stratum granulosum. In the basal cells, only early gene expression occurs, and these cells allow the persistence of PV infection. Viral protein (L1 and L2) synthesis and virion assembly occur in terminally differentiated cells of the stratum spinosum and, more specifically, the stratum granulosum. The virus is present in the differentiated keratinized cells and is shed with exfoliated cells of the stratum corneum (Figure 2).

Due to their ability to alter cell regulation, PVs can also induce tumours. PV is one of the most common causes of viral neoplasia in humans and has also been associated with neoplasia in dogs, horses, ruminants and pigs. Both E6 and E7 are considered oncogenes, expressing proteins that inhibit the retinoblastoma (pRB) and p53 protein, respectively, resulting in interference with normal cell regulation leading to malignant lesions [25]. PV is, however, commonly found in the healthy skin of different animals, including the cat [26]; this makes definitive proof of a causal relationship between the presence of PV sequences and skin lesions difficult.

## 5. Immunity

Papillomaviruses tend to stimulate a mild immune response, due to viral replication in the superficial layers of the epithelium. If an effective immune response is induced, it is based on cell-mediated immunity [4,27]. This leads to the lysis of infected cells and the resolution of the hyperplastic lesions. Antibodies can be induced but do not appear to play a role in lesion resolution. However, antibodies can play a role in the type-specific protection against (re)infection [28].

## 6. Clinical Signs

The majority of cats are infected by PVs, but the disease seems to occur less frequently compared to other domestic animals [4,29]. Nevertheless, several diseases have been associated with different types of PVs (Table 1). Lesions reported to be associated with PVs in domestic cats include hyperplastic plaques, skin papillomas, oral papillomas, Bowenoid in situ carcinomas (BISCs), cutaneous and oral squamous cell carcinomas ((O)SCCs), feline basal cell carcinomas and feline sarcoids. Also, there is evidence for the involvement of FcaPV2 in the development of MCC in cats (Table 2) [30].

### 6.1. Hyperkeratotic Plaques

**Figure 3:**
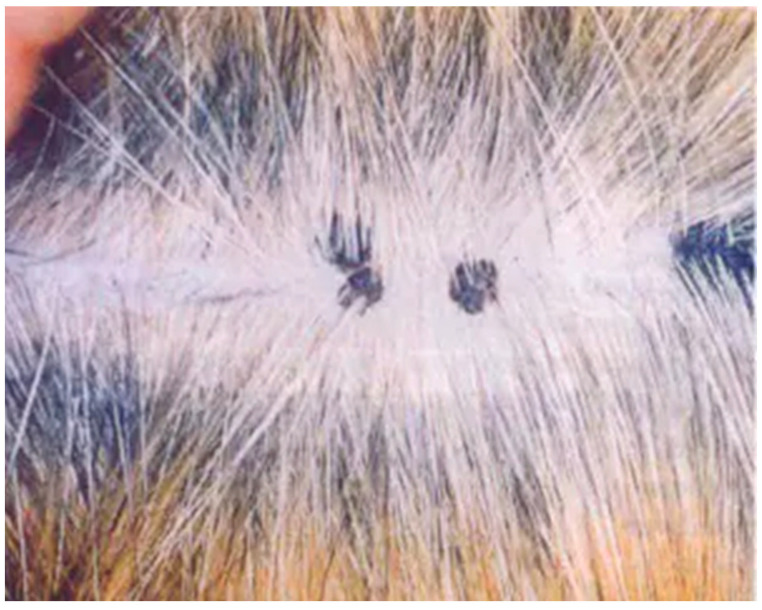
Pigmented flat cutaneous papillomas (photo Herman Egberink, Ph.D thesis Utrecht).

Cutaneous hyperkeratotic plaques seem to be most common in older and immunosuppressed cats, e.g., FIV-infected animals [31,32]. However, plaques have also been reported in cats without any recognised immunodeficiency [33]. The plaques appear as flat, slightly raised, scaly and variably pigmented lesions and are generally less than 1 cm in diameter (Figure 3).

Feline catus PVs 2, 3 and 5 have been associated with viral plaques [34,35]. The lesions share many histological features with BISCs, and are thought to be precursors of BISC, representing different severities of the same disease [33].

### 6.2. Skin Papillomas (Warts)

Papillomas of the skin, characterised by marked thickening and folding of the epidermis, seem to be rare in cats, in contrast to dogs and other domestic animals. To date only three cases of PV-induced cutaneous papillomas have been reported in domestic cats. In one cat with a focal area of hyperplasia on the eyelid, PV L1 was detected in the skin lesion by immunostaining [36]. In a cat with a papilloma that developed on the nasal planum only, sequences from human PV type 9 could be amplified [37]. PVs are usually highly species-specific, and detection of these human PV-type sequences might be due to cross-contamination and not cross-infection. Humans are ubiquitously and asymptomatically infected, which can lead to sample contamination. The third case was a 4.5-year-old male domestic shorthair cat with a papilloma on the nasal planum. The PV DNA sequence amplified from the lesion had a low similarity to other feline PV types, suggestive of a novel PV genus [7].

### 6.3. Oral Papillomas

Only a few cases of oral papillomas have been described in cats; they seem to occur less frequently than in other species [38]. However, because these papillomas seem to be restricted to the ventral surface of the tongue, it was suggested that they might occur more frequently but remain undetected [4]. The lesions appear as clusters of exophytic lesions on the ventral surface of the tongue. They are characterised histologically by foci of markedly hyperplastic folded epithelium with PV-induced cell changes. In a report describing two cats, oral papillomas were associated with FcaPV1 [38]. This feline PV type is the only feline type classified within the genus *Lambdapapillomavirus*, in which the PV types that cause oral papillomas in dogs and some other species also belong [4].

### 6.4. Bowenoid In Situ Carcinomas (BISCs)

Feline BISCs are superficial lesions that are confined to the epidermis and occur often in pigmented-haired skin as crusting, de- or hyperpigmented, and roughly circular lesions [33]. BISCs tend to be larger than viral plaques and are more markedly raised [39]. The lesions are typically found in middle-aged or older cats [33]. Spontaneous regression can occur, although many BISCs persist, and some progress to an invasive squamous cell carcinoma (ISCC). Devon Rex and Sphynx breeds seem to be more prone to developing BISC; lesions develop at an earlier age and tend to be more aggressive, rapidly progressing to ISCC and metastatic SCC [40,41]. A clear association between PV DNA (the then-called *Felis domesticus* papillomavirus 2; FdPV-2) and feline SCCs has been found; viral DNA was detected in all 20 BISCs examined and in 17 out of 20 cases of SCCs [42]. However, FdPV-2 DNA was also detected in 52% of healthy skin swabs [26]. Although FdPV-2 has been detected most frequently in BISCs and SCCs, other PV types and FcaPV3, FcaPV4 and FcaPV5 were identified as well [14,43,44].

### 6.5. Feline Cutaneous Squamous Cell Carcinomas (SCCs)

Cutaneous SCCs are common skin cancers in cats. Sunlight plays a role in their development. Lesions tend to be found in sparsely haired areas such as the eyelids, nose, and pinnae and typically occur in non-pigmented skin. A contributory role for FcaPV is suggested by the more frequent detection of FcaPV2 DNA in cutaneous SCCs than in the healthy skin of cats [42,45]. The relative roles of ultraviolet (UV) light, other co-factors, or FcaPV infection in the development of SCCs are uncertain [4]. Studies suggest that FcaPVs could have an aetiological role in a quarter to a third of all cutaneous SCCs [45,46,47]. Besides the more frequent detection of FcaPV2 in SCC lesions, other studies support its role in the development of SCCs. Firstly, higher viral loads have been detected in BISCs and in a subset of SCCs than in healthy skin [48]. Secondly, FcaPV2 RNA, as evidence of gene expression, has been detected in a proportion of SCCs but not in healthy skin [25,48]. Also, staining of the p16CDKN2A protein can be visualised in SCCs that also contain PV DNA (see “diagnosis” section later) [49,50]. Feline PV type 2 is the most common type of PV detected in cutaneous SCCs, but in some cases of SCCs, FcaPV3, FcaPV4 or FcaPV6 DNA has been identified [44,51,52]. In one study, 50% of the sequenced PV DNA was most closely related to human PV DNA [18].

### 6.6. Oral Squamous Cell Carcinomas (OSCCs)

Feline oral SCCs are aggressive invasive neoplasms and an important cause of mortality [4,46]. They are most commonly located in the sublingual/lingual region, maxilla, mandible, buccal mucosa, lip and caudal pharynx/tonsillar region [53]. In humans, PV is an established cause of a proportion of the OSCCs. Consequently, several studies on the aetiology of feline OSCCs have focused on the detection of PV in OSCC lesions. Results of these studies show significant differences in the detection of PV DNA within feline OSCCs, as described below.

Papillomavirus DNA could not be detected in any of the 30 cases of OSCC from cats in New Zealand nor in seven OSCC samples screened in cats from Japan [52,54]. In cats in New Zealand, FcaPV1 DNA was found in only one of 36 samples, and also in one of 16 samples from cats with non-neoplastic inflammatory lesions [55]. Recently, using a metagenomic approach for DNA viruses, the virome was sequenced and FcaPV3 was detected in only one of 20 OSCC samples and none of 9 samples of healthy gingiva [56]. These findings contrast with the results of other studies. In a study performed in Italian cats, a higher number of samples from cats with OSCC and non-neoplastic ulcerative oral lesions were positive for FcaPV2 DNA, namely 10 of 32 (31%) and 4 of 11 (36%), respectively [57]. The presence of viral gene expression suggested active viral replication, but no significant differences in viral loads were detected between the samples of OSCC and ulcerative oral lesions. In a recent study, 11 of 19 (57.9%) OSCC samples of cats from Taiwan and one of 9 (11.1%) OSCC samples of cats from Japan contained FcaPV2 DNA, but no oral samples from cats without OSCC samples were included [58]. In conclusion, the markedly different results for the detection of numbers of PV-positive OSCC samples together with similar detection rates in non-neoplastic samples seem, so far, not to support a role for PV in the development of OSCCs although it is acknowledged that FcaPV2 is most commonly identified.

### 6.7. Feline Basal Cell Carcinomas (BCC)

Basal cell carcinomas are rare feline skin neoplasms presenting mostly as single ulcerated raised plaques or nodules; keratinization is generally absent [46]. An association between PV infection and feline BCC was suggested by the presence of PV cytopathic changes in cells within BCCs [4]. The association was further supported by the detection of FcaPV3 DNA in a cat with multiple BCCs [43] and the amplification of sequences from a feline BCC of a probably novel type of PV that was not further classified [59]. A novel type was amplified from a BCC, and was designated FcaPV7 and considered to be associated with skin cancer. The histological lesions were different from those reported with other subtypes of FcaPV [15].

### 6.8. Feline Cutaneous Fibropapillomas or Feline Sarcoids

Feline sarcoids are rare, presenting as skin neoplasms; solitary or multiple nodular masses are found most commonly on the head (nasal philtrum, lips), neck, ventral abdomen and limbs.

Lesions are characterised by the proliferation of mesenchymal rather than epithelial cells. Metastasis does not occur [60]. The finding of a PV similar to BPV type 14 (BPV14) and the higher prevalence in cats with known exposure to cattle suggest an association with the bovine virus [16,61,62]. Also, PV DNA could be localised in neoplastic mesenchymal cells by in situ hybridisation (ISH) [62]. This hypothesis is in line with the known association between BPV and equine sarcoids [63].

### 6.9. Merkel Cell Carcinoma (MCC)

Merkel cell carcinoma is a rare and aggressive neuroendocrine carcinoma of the skin. In humans, Merkel cell polyomavirus (MCPyV) is involved in approximately 80% of Merkel cell carcinomas, and in 20% of cases, UV light exposure plays a role [64]. Tumorigenesis is caused by the integration of the MCPyV DNA into the host genome and the induction of persistent expression of viral oncogenes or through the occurrence of DNA mutations induced by UV exposure [64,65]. Feline MCCs are rare skin tumours, located in the dermis and/or subcutis and present as firm, red, dome-shaped, solitary skin nodules. The overlying skin is frequently ulcerated [66]. The tumours are characterised by the expression of some epithelial markers (cytokeratin 18 and 20) and neuroendocrine markers, such as synaptophysin and CD56 [66]. Cats with MCC often have other proliferative skin lesions such as SCC and BCC, and a role for FcaPV2 in feline MCC has also been suggested [30]. FcaPV DNA could be detected in 95% of feline MCC cases and the presence of FcaPV DNA in the tumour cells was confirmed by ISH [30]. The hybridisation signal pattern suggested the integration of the DNA into the genome, which was further evidenced by whole genome sequencing of two FcaPV-positive cases [11].

**Table 1:** Papillomavirus lesions and their associated papillomavirus type.

Papillomavirus Lesion	Papillomavirus Type	Reference
Hyperkeratotic plaques	FcaPV2, * FcaPV3, FcaPV5	[35] [40] [67]
Skin papillomas	HPV9 ? ** FcaPV, type not classified	[37] [7]
Oral papillomas	FcaPV1	[38]
Bowenoid in situ carcinomas	FcaPV2, FcaPV3, FcaPV4, FcaPV5	[42] [14] [44]
Feline cutaneous squamous cell carcinomas	FcaPV2, FcaPV3, FcaPV4, FcaPV6	[42] [52] [51,58]
Oral squamous cell carcinomas	FcaPV2 ?	[57]
Feline basal cell carcinoma	FcaPV3, FcaPV7 ?	[43] [15]
Feline sarcoids	BPV14 ***	[26]
Merkel cell carcinoma	FcaPV2	[11,30]

* FcaPV indicates Feline catus papillomavirus; ** HPV indicates human papillomavirus; *** BPV indicates bovine papillomavirus; “?” indicates results of studies vary or are unclear.

**Table 2:** Papillomavirus lesion and clinical presentation.

Lesion	Clinical Signs	Prognosis/Treatment
Hyperkeratotic plaques	Flat, slightly raised, scaly and variably pigmented lesions.	Spontaneous regression possible but some might persist. No specific treatment.
Skin papillomas	Localised lesions: thickening and folding of the epidermis.	No specific treatment. Lesions can be removed surgically. Recurrence is possible (as in dogs with canine papillomas).
Oral papillomas	Exophytic lesions on the ventral surface of the tongue.	Incidental occurrence. No clinical signs. Most probably resolve spontaneously.
Bowenoid in situ carcinomas	Crusting, mostly hyperpigmented and roughly circular lesions, often in pigmented, haired skin.	Spontaneous regression can occur. But also progression to ISCC (metastasis especially reported in Devon Rex and Sphynx cats). Surgical excision, cryo-surgery or carbon dioxide laser surgery can be considered. Efficacy and safety of imiquimod (used in humans) needs additional controlled studies.
Feline cutaneous squamous cell carcinomas	Majority present in non-haired, non-pigmented areas of the body.	PV-associated SCCs have a more favourable prognosis than non-PV induced.
Oral squamous cell carcinomas	Exophytic filiform masses on the ventral surface of the tongue.	Almost all invariably fatal.
Feline basal cell carcinoma	Mostly single, ulcerated raised lesions, with keratinization generally absent.	Lesions can be removed surgically.
Feline sarcoids	Solitary or multiple nodular masses found most commonly on the head (nasal philtrum, lips), neck, ventral abdomen and limbs.	Often recur after surgical removal, but metastasis does not occur.
Merkel cell carcinoma	Firm, red, dome-shaped, solitary skin nodules; overlying skin is frequently ulcerated.	Poor prognosis. Lesions can be removed surgically but often recur, and tendency for metastasis (despite margin-negative surgery).

ISCC; invasive squamous cell carcinoma.

## 7. Diagnosis

**Figure 4:**
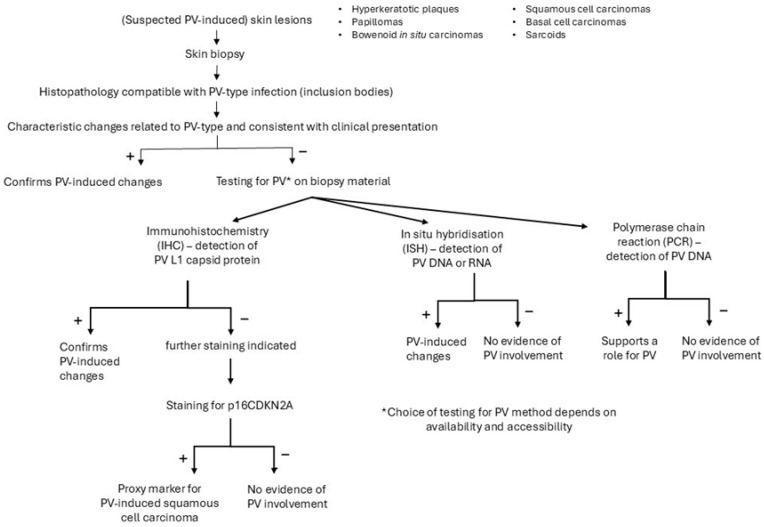
Diagnostic procedure to support the role of a papillomavirus (PV) infection in case of a clinical suspicion of a PV-induced lesion.

Several methods can support the diagnosis of a viral-induced hyperplastic lesion (Figure 4). A biopsy from a skin lesion can be taken for histopathologic examination and immunohistochemical staining of PV group-specific antigens [4]. The different PV-induced lesions of virally-induced oral papillomas, BISCs and feline sarcoids all show characteristic histological changes, often related to the PV type involved [4]. Infection of cells with FcaPV1 can result in eosinophilic intracytoplasmic bodies [38], infection with FcaPV2, in expanded cytoplasm by clear or slightly granular grey-blue material, and with FcaPV3, slender elongated perinuclear basophilic bodies [68]. In cells infected with FcaPV4 or FcaPV5, the cytoplasm may be expanded by dark blue-grey material [67,69]. These changes become less visible in advanced neoplastic lesions where there is less, or no, viral replication. In lesions with PV-induced cell changes, antibodies against the L1 protein can be used to stain the PV protein within cells. However, feline PV-type specific antibodies are not commercially available and antibodies against PV types of other species (including humans) might not cross-react; if the cross-reactivity with feline-type PVs is unknown, a negative result will be difficult to interpret [4]. Immunostaining is of no use in PV-induced cancers since viral replication in these lesions is rare. However, in these lesions, immunostaining of p16CDKN2A protein (p16) can be performed as a proxy marker for PV-induced SCCs [70]. The p16 inhibits the retinoblastoma protein (pRB) to prevent cell division. In PV-induced neoplasms, the pRB is degraded, which leads to the loss of pRB and increased amounts of p16 [4]. A strong association between the presence of PV-DNA and p16 immunostaining has been shown [45,48]. Also, PCR can be used to demonstrate PV DNA in the lesions and to identify the viral strain by further sequencing. However, the presence of PV DNA in the healthy skin of cats makes interpretation of positive PCR results of skin lesions difficult. Therefore, a positive PCR result is not definitive proof that the lesion is caused by PV if DNA is extracted from the whole sample. In contrast to PCR, ISH detects PV DNA or RNA within the cells of a lesion, and the presence of PV DNA or RNA in the basal and suprabasal epithelial layers of the lesion supports the role of the virus in the pathogenesis [4].

## 8. Treatment

No specific treatment for feline PV-induced skin lesions is known. In immunocompetent cats, spontaneous regression can be expected, similar to dogs, but it might take up to several months. The treatment of superficial layers of the skin can be sufficient for lesions that are confined to the epidermis, like BISCs. Imiquimod cream stimulates inflammation and is used for the topical treatment of Bowen’s disease in humans. The cream has never been thoroughly evaluated in cats with this condition; although a response was noted, no conclusion with respect to the efficacy of the drug in cats was reached in one study [71]. In this study, the SSC lesions were also PV antigen-negative. Although anecdotal evidence supports the use of this medication, more controlled studies are required to evaluate the safety and efficacy of this treatment. If only a few superficial lesions are present, surgical excision can be considered assuming that all of the affected epidermis can be removed [4]. Carbon dioxide laser therapy, which has been used in other species, can also be considered [72], particularly in areas where wound closure is difficult. Cryotherapy can also be used to treat the superficial lesions [4]. Feline ISCCs tend to slowly metastasise. Therefore, if anatomical location allows, complete excision might be curative.

## 9. Prognosis

Most PV infections remain asymptomatic with limited replication in epithelial cells. Cutaneous and oral papillomas often resolve spontaneously. The prognosis of PV-induced SCCs depends on the number, location and invasiveness of the lesions. PV-induced SCCs tend to have a more favourable prognosis than non-PV-associated SCCs [60]. If successfully treated, e.g., by excision of the lesion, new additional lesions might develop in the same cat. Sphinx and Devon Rex cats are predisposed to BISCs and have a higher risk of developing invasive and metastatic SCCs [40]. Therefore, all viral plaques/BISCs should be carefully monitored for progression to SCC. Feline OSCCs are almost invariably fatal, but no association of feline OSCCs with PV infection has been proven.

## 10. Vaccination

Although vaccination against PV infections can be effective, as evidenced by the use of successful vaccines in humans, no vaccines are available for papillomatosis in cats. In one study, an experimental FcaPV2 virus-like particle vaccine was shown to be safe and immunogenic [73], but vaccination-induced high antibody titres had no effect on the viral loads in cats that were already infected [73]. The majority of PV-induced lesions in cats are caused by FcaPV2, which infects cats within the first few days of life [74]. To prevent disease development, a FcaPV2 vaccination would need to be given before infection, which is not feasible due to early infection. Vaccines against other PV types are unlikely to be developed, considering the development costs and the relatively rare occurrence of PV-induced disease by these virus types [4].

## Data Availability

No new data were created or analysed in this study. Data sharing is not applicable to this article.
